# Differential Expression and Function of Bicellular Tight Junctions in Skin and Oral Wound Healing

**DOI:** 10.3390/ijms21082966

**Published:** 2020-04-23

**Authors:** Trevor R. Leonardo, Junhe Shi, Dandan Chen, Harsh M. Trivedi, Lin Chen

**Affiliations:** 1 Department of Microbiology and Immunology, College of Medicine, University of Illinois at Chicago, Chicago, IL 60612, USA; tleona3@uic.edu; 2 Center for Wound Healing and Tissue Regeneration, College of Dentistry, University of Illinois at Chicago, Chicago, IL 60612, USA; jshi36@uic.edu; 3 Colgate-Palmolive Company, Piscataway, NJ 08854, USA; dandan_chen@colpal.com (D.C.); Harsh_M_Trivedi@colpal.com (H.M.T.)

**Keywords:** tight junction, skin, oral mucosa, epithelium, keratinocytes, wound healing

## Abstract

Bicellular tight junctions are multiprotein complexes that are required for maintenance of barrier function and fence function in epithelial tissues. Wound healing in the oral cavity leads to minimal scar formation compared to the skin, and the precise mechanisms for this regenerative response remain to be elucidated. We hypothesized that oral and skin tissues express a different tight junction repertoire both at baseline and during the wound healing response, and that these molecules may be critical to the differential repair between the two tissues. We re-analyzed a mouse skin and palate epithelium microarray dataset to identify the tight junction repertoire of these tissue types. We then re-analyzed a skin and tongue wound healing microarray dataset to see how expression levels of tight junction genes change over time in response to injury. We performed in vitro scratch assays on human oral and skin keratinocyte cell lines to assay for tight junction expression over time, tight junction expression in response to lipopolysaccharide and histamine treatment, and the effects of siRNA knockdown of claudin 1 or occludin on migration and proliferation. Our data showed that oral and skin epithelium expressed different tight junction genes at baseline and during the wound healing response. Knockdown of claudin 1 or occludin led to changes in proliferation and migration in human skin keratinocytes but not oral keratinocytes. Furthermore, we also showed that skin keratinocytes were more permeable than oral keratinocytes upon histamine treatment. In conclusion, this study highlights a specific subset of functional tight junction genes that are differentially expressed between the oral and skin tissues, which may contribute to the mechanisms leading to distinct healing phenotypes in response to injury in the two tissues.

## 1. Introduction

Wound healing is a complex process that occurs across four overlapping stages: hemostasis, inflammation, proliferation, and remodeling. These stages involve multiple cell types that are required to repair and regenerate the affected tissue including fibroblasts, keratinocytes, adipocytes, endothelial cells, and various immune cells. In addition, factors produced by these cells, including cytokines, chemokines, growth factors, and anti-microbial peptides, play important roles in the normal healing process [1]. Comparisons of the skin to specific regions of the oral cavity, such as the hard palate, the anterodorsal surface of the tongue, and the gingiva have shown that the epidermis/epithelium and dermis/lamina propria of these tissues are histologically and intrinsically different [2]. Furthermore, the embryologic origins of skin and epithelial regions of the oral cavity vary as well. Some histological differences include the presence of hair follicles, sweat glands, and sebaceous glands in skin, while the tongue has taste buds. Despite these differences, both skin and oral tissues are regularly exposed to hazardous conditions such as allergens, poisons, injury/trauma (lacerations or surgical procedures), pathogens (bacteria, viruses and parasites) or temperature changes, which can result in disruption of tissue homeostasis and lead to pathologies. Previous studies have shown that wounds in the oral cavity heal more rapidly with significantly less inflammation, decreased hypoxia, faster re-epithelialization, a more refined angiogenic response and minimal scar formation [3,4,5,6,7]. Although many factors are responsible for the differential healing outcomes between these two tissues including dissimilar environments and structural variations, intrinsic differences in the unwounded tissues and their differential transcriptomic response to injury are probably the core factors [3,4,8,9,10].

Bicellular tight junctions (TJs) are multiprotein complexes composed of more than 40 different molecules. Integral transmembrane protein families include claudins (CLDN), junctional adhesion molecules (JAM), occludin (OCLN), and the MAL and related proteins for vesicle trafficking and membrane link (MARVEL) family, such as MarvelD3 [11,12,13,14]. In addition, there are peripheral intracellular membrane proteins that connect transmembrane TJ molecules and actin filaments such as the zonula occludens (ZO) proteins [11,12,13]. TJ genes appear to be expressed to some degree in most tissues. TJ molecules from neighboring cells form paired strands to seal the paracellular pathway. Regulation of ions and molecules passing through these junctions is based on their charge and size selectivity. This is one of two major biological functions of TJs called barrier function. TJs also maintain cell polarity and prevent mixing of molecules from the apical and lateral membranes, a role termed fence function. In addition, some TJ genes also regulate the proliferation and migration of epithelial cells [15,16] and are involved in the immune response [17]. Given the differential healing phenotypes of cutaneous skin and oral wounds and the critical roles that TJs play in various tissues, we hypothesized that the repertoire of expressed TJ genes may be different between the skin and oral cavity in both intact epithelial cells and in a wound healing setting. Such differences could contribute to mechanisms that lead to the distinct healing phenotypes of skin and oral response to injury. We re-analyzed microarray data of normal mouse skin epithelia and hard palate epithelia to identify differences in expression of TJ genes. We also identified several TJ genes that are differentially expressed in the skin and tongue wound healing response in mouse. We further demonstrated that human skin keratinocytes and gingival keratinocytes respond differently in their expression of certain TJ genes in response to injury and lipopolysaccharide (LPS) stimulation. Finally, inhibition of claudin 1 or occludin altered the migratory and proliferative capacity of human skin but not oral keratinocytes.

## 2. Results

### 2.1. Differential Expression of TJ Molecules in Primary Mouse Skin and Oral Epithelial Cells

**Figure 1:**
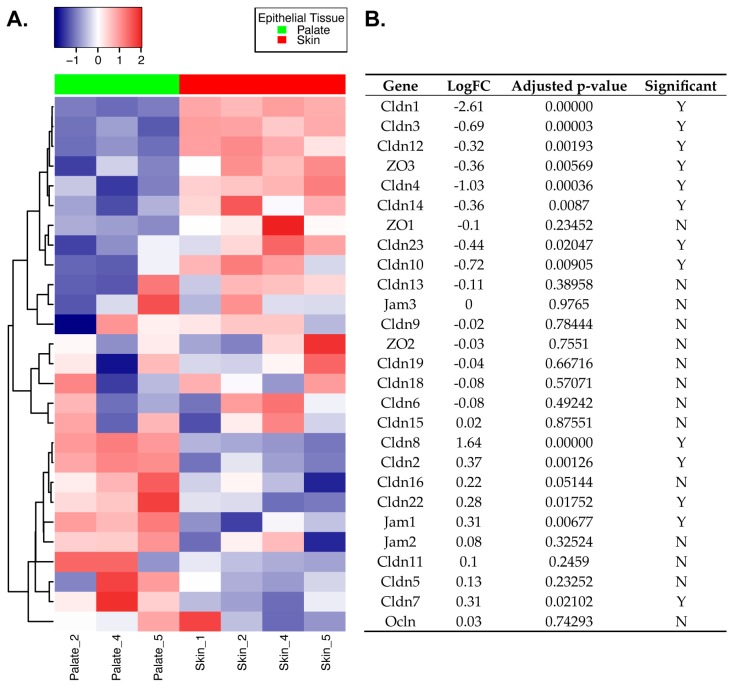
Differential expression of tight junction molecules in primary mouse skin and oral epithelial cells. Skin epithelium obtained from the tails of mice were compared to oral epithelium obtained from the hard palate of mice using the R/Bioconductor package linear models for microarray data (limma). (**A**) Heatmap displaying gene expression profiles of the 27 tight junction (TJ) genes analyzed. Genes were hierarchically clustered using the Euclidean distance measure and complete clustering, with gene expression scaled across rows. Each row is a gene and each column is a sample. (**B**) Table of limma results for the corresponding 27 TJ genes analyzed. The gene column is aligned to match and annotate the corresponding heatmap in A (left). LogFC is the calculated log_2_ fold change between oral and skin epithelium, with positive values corresponding to over expression in palate and negative values corresponding to over expression in skin. Adjusted *p*-value is listed in the second column, and the third column identifies whether or not the gene was significantly differentially expressed (Y = yes, N = no) using an adjusted *p*-value of *p* < 0.05. *n* = 4 mice for skin and *n* = 3 mice for palate epithelium. Data from one of the palate samples (Palate_1) was excluded in this analysis due to its poor quality (see the Materials and Methods section).

An integral component of skin and oral tissue homeostasis is barrier function. Although both tissues use tight junctions for various aspects of barrier function, the level of similarity of their TJ repertoire is unknown. To investigate this, we performed a differential expression analysis on a dataset that was previously generated in our lab (GSE56135). This dataset includes epithelium from mouse hard palate and skin isolated by enzymatic disassociation from the underlying lamina propria and is therefore primarily made up of keratinocytes from each respective tissue. We used the R/Bioconductor software package linear models for microarray data (limma) to compare the mouse gene expression profiles of three palate epithelium samples to four skin epithelium samples and set our significance cutoff at an adjusted *p*-value of *p* < 0.05 using the Benjamini and Hochberg (BH) method. We then subset the differential expression results to look specifically at 27 TJ genes, including claudin 1–23, jam 1–3, tight junction protein 1–3 (*ZO1, ZO2, ZO3*), and occludin, and visualized the results (Figure 1A and Figure S1). Claudin 17, 20, and 21 were not analyzed as they did not meet chip description file (CDF) probe mapping criteria (see the Materials and Methods section). Using the adjusted *p*-value cutoff above, 13/27 (48%) TJ genes were differentially expressed between the two tissue epithelia, with eight genes over expressed in skin compared to palate (*Cldn1, Cldn3, Cldn4, Cldn10, Cldn12, Cldn14, Cldn23, ZO3*) and five genes over expressed in palate compared to skin (*Cldn2, Cldn7, Cldn8, Cldn22, Jam1*) (Figure 1B). *Cldn1 and Cldn8* were the most differentially expressed TJ genes between the two tissues, with *Cldn1* having significantly higher expression with a log_2_ fold change (logFC) of 2.61 in skin epithelium and *Cldn8* being significantly higher in palate epithelium (logFC 1.64). *ZO3* was slightly over expressed in the skin compared to palate (logFC 0.36), and *Jam1* was over expressed in the palate (logFC 0.31). These results demonstrate that the palate and skin epithelium may utilize different TJ genes to maintain barrier function. Having identified a substantial number of TJ genes that are differentially expressed at baseline in the two tissue epithelia, we next wanted to explore the relationship between the expression of TJ genes when barrier integrity is compromised in a wound healing setting.

### 2.2. Differential Expression of TJ Molecules in Mouse Skin and Oral Wounds In Vivo

**Figure 2:**
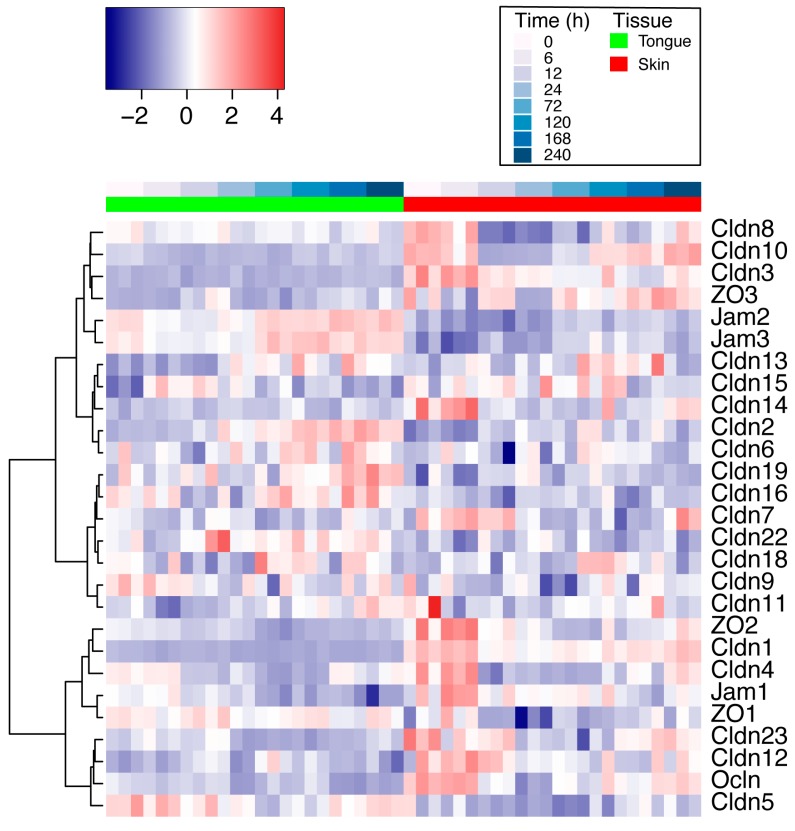
Differential expression analysis of tight junction molecules in mouse skin and oral wounds in vivo. A heatmap displaying the gene expression profiles of the 27 TJ genes analyzed in response to wounding over 10 days in both skin and tongue wounds. Genes were hierarchically clustered using the Euclidean distance measure and complete clustering, with gene expression scaled across rows. Each row is a gene and each column is a sample at a specific time point. Time 0 represents baseline expression in normal tissue, and subsequent time points represent hours after wounding. Samples are displayed in response to injury over time, going left to right for each tissue using a white–blue color scale bar, with tissues denoted by green or red color bars. *n* = 3 mice per group per time point for both skin and oral wounds.

To investigate the TJ gene expression response to wounding in skin and oral tissues, we re-analyzed a previously generated microarray dataset from our lab that spans the first 10 days of the wound healing process in mouse skin and tongue (GSE23006). It is important to note that this study performed whole tissue excisional biopsies and therefore this analysis includes cell types from the epithelium and from the respective sub-epithelial tissue layers below for both the skin and tongue. We used limma to perform the following three comparisons: (1) response to wounding over time in mouse skin; (2) response to wounding over time in mouse tongue; (3) differential response to wounding over time between mouse skin and tongue. The same subset of 27 TJ genes as used above were examined in this dataset after the initial differential expression comparisons were performed, and gene expression profiles were visualized by heatmap (Figure 2). Statistical significance was defined as an adjusted *p*-value (*p* < 0.05) using the BH method. For the first comparison, 19/27 (70%) of the TJ genes were significantly differentially expressed in at least one time point in response to wounding in the skin (Table 1). There were 4/27 (15%) genes that were upregulated (*Cldn2, Cldn13, Cldn18, Jam1*) in response to wounding in skin when compared to baseline tissue expression, 10/27 (37%) TJ genes downregulated (*Cldn3, Cldn5, Cldn8, Cldn10, Cldn11, Cldn14, Cldn23, Jam2, Ocln, ZO3*), and 5/27 (18%) both up- and downregulated at various time points (*Cldn1, Cldn4, Jam3, ZO1, ZO2*). Interestingly, *Cldn1, Cldn4, ZO1,* and *ZO2* were upregulated initially at 6 h, followed by subsequent downregulation at various later time points when compared to baseline expression. In our second comparison, 11/27 (41%) of the TJ genes were differentially regulated in response to wounding in at least one time point in the tongue (Table 2). We found 3/27 (11%) TJ genes to be upregulated (*Cldn2, Cldn13, Cldn15*) and 7/27 (26%) downregulated (*Cldn4, Cldn5, Cldn10, Jam1, Jam2, Ocln, ZO2*), and found *Jam3* to be both up- and downregulated over time when compared to baseline expression levels. In the third comparison, we identified 22/27 (81%) of the TJ genes as being differentially expressed between skin and tongue in at least one time point in response to wounding (Table S1). *Cldn5, Jam2, and Jam3* were upregulated at all time points in tongue compared to skin. However, upon further inspection, their temporal expression profiles were similar between the two tissues (Figure S2). In contrast, *Cldn1, Cldn3,* and *Cldn10* were more highly expressed at most time points in skin compared to tongue, highlighting their potential tissue specificity. *ZO1, ZO3*, and *Ocln* also had differential healing responses in skin and tongue and were therefore included in subsequent in vitro analyses. Taken together, these results demonstrate that TJ genes in oral and skin tissues are differentially regulated in response to wounding, suggesting that TJ composition may play a role in the differential healing response between these tissues.

### 2.3. Differential Expression of TJ Molecules in Human Skin and Oral Keratinocytes after Injury

**Figure 3:**
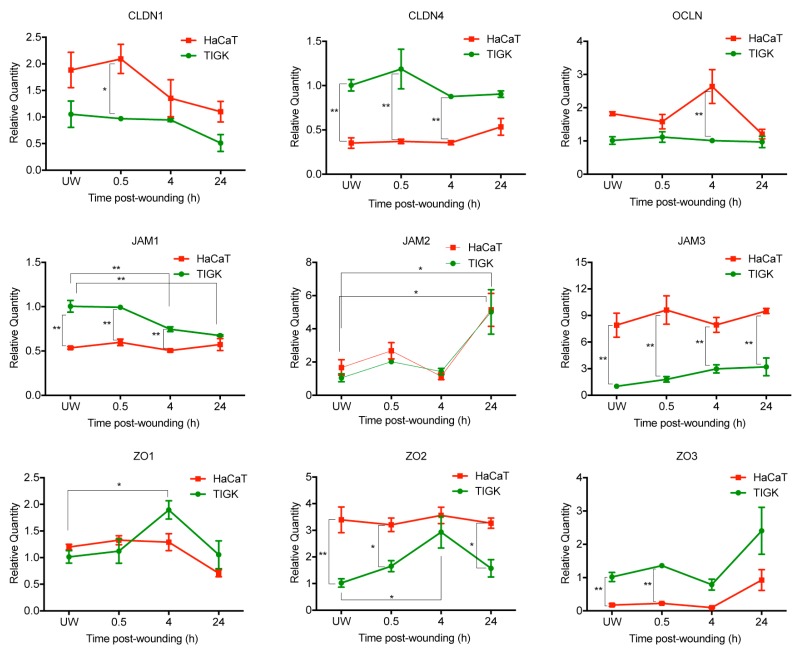
Differential expression of tight junction molecules after injury in skin and oral keratinocytes. Immortalized human skin keratinocyte (HaCaT) and human gingival keratinocyte (TIGK) cells were cultured in a 12-well culture plate. When the cells were confluent, three horizontal and three vertical scratch wounds were made. mRNA expression of claudin *(CLDN) CLDN1, CLDN4*, occludin *(OCLN)*, junctional adhesion molecule *(JAM) JAM1, JAM2, JAM3*, zonula occludens *(ZO) ZO1, ZO2, and ZO3* were examined 0.5, 4, and 24 hours (h) after wounding by real-time PCR. UW: unwounded. *n* = 3 replicates, * *p* < 0.05, ** *p* < 0.01.

Keratinocytes are the main cell type in the epithelium of skin and oral mucosa, and expression of various TJ genes in these cells is critical for maintenance of barrier function. To identify differences between human skin and oral keratinocytes, we took an in vitro approach to examine the mRNA expression levels of a regularly studied subset of the 27 previously examined TJ genes in keratinocytes at baseline or post-injury. Monolayers of human skin keratinocytes (HaCaT) and human gingival keratinocytes (TIGK) were injured by pipette tip scratch assay followed by RT-PCR analysis of TJ genes including *CLDN1, CLDN4, JAM1-3, OCLN*, and *ZO1-3* (Figure 3). *CLDN4, JAM1*, and *ZO3* had higher expression levels in TIGK at baseline and after injury when compared to HaCaT, while *CLDN1, OCLN, JAM3*, and *ZO2* were more highly expressed in HaCaT at baseline and after injury. *JAM1* was repressed after injury in TIGK cells at both 4 and 24 h, whereas *ZO1* and *ZO2* were transiently upregulated at 4 h. *JAM2* and *ZO3* followed similar patterns of expression in both HaCaT and TIGK cells in response to injury, with statistically significantly increased expression of *JAM2* after 24 h compared to unwounded expression. These results demonstrate a potentially conserved response to injury through *JAM2* and *ZO3* expression changes, and an oral-specific response to injury through *JAM1, ZO1*, and *ZO2* gene expression changes. 

### 2.4. Differential Expression of TJ Molecules in Human Skin and Oral Keratinocytes after LPS Treatment

**Figure 4:**
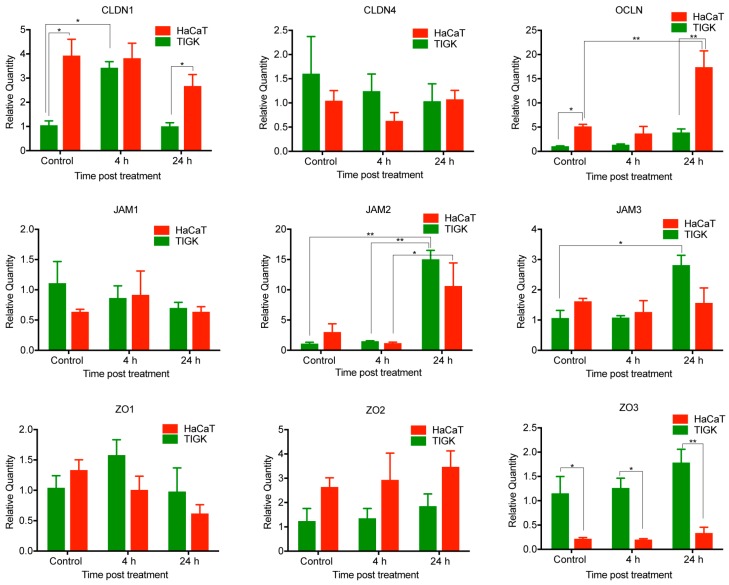
Differential expression of tight junction molecules after lipopolysaccharide (LPS) treatment in skin and oral keratinocytes. HaCaT and TIGK cells were cultured in a 12-well culture plate. The cells were treated with LPS (100 ng/mL) when reaching 80% confluency. mRNA expression of *CLDN1, CLDN4, OCLN, JAM1, JAM2, JAM3, ZO1, ZO2*, and *ZO3* was examined at 4 and 24 h after treatment by Real-Time PCR. Control: untreated. *n* = 3 replicates, * *p* < 0.05, ** *p* < 0.01.

Both skin and oral keratinocytes are constantly exposed to bacteria in living organisms. We wanted to investigate if the mRNA expression of TJ molecules in HaCaT and TIGK would be altered over time after exposure to LPS, a characteristic component of the cell wall of gram-negative bacteria. To test this, HaCaT and TIGK cells were grown to 80% confluency, then treated with LPS and subjected to RT-PCR analysis for a subset of TJ genes at 4 and 24 h (Figure 4). *CLDN1* was transiently upregulated as early as 4 h in TIGK cells in response to LPS treatment and returned to baseline by the 24 h time point, whereas expression in HaCaT cells was not altered at any time point. Expression levels of *JAM2* and *JAM3* were statistically significantly upregulated at the 24 h time point in TIGK cells, but not HaCaT. However, the expression trend over time of *JAM2* in TIGK and HaCaT was similar, which also parallels the observed expression changes in the in vitro scratch assay. Occludin was over expressed at 24 h after LPS treatment in HaCaT cells compared to control, but not in TIGK. On the basis of these results, there is a differential response in gene expression levels in LPS-treated skin and oral keratinocytes in vitro. Finally, there is also a conserved response between both oral and skin keratinocytes in response to wounding and LPS treatment through expression changes in *JAM2*.

### 2.5. Inhibition of Claudin 1 or Occludin Alters Migration and Proliferation/Viability of Skin Keratinocytes but Not Oral Keratinocytes

**Figure 5:**
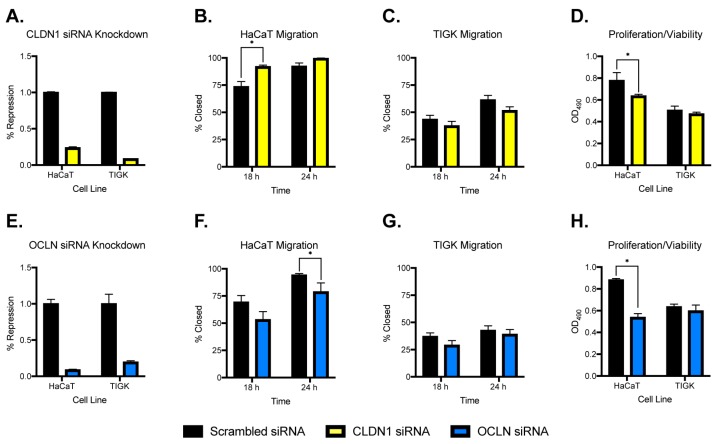
Migration and proliferation/viability of skin and oral keratinocytes after *CLDN1* or *OCLN* knockdown. For migration assays, HaCaT and TIGK cells were transfected with claudin 1 or occludin small interfering RNA (siRNA). The transfected cells were cultured in 2-well silicone culture inserts with a defined cell-free gap as described in the Materials and Methods section. Cell migration was documented by a digital camera 18 and 24 h later. For proliferation/viability assays, 5 × 10^3^ cells/well of claudin 1 or occludin siRNA-transfected HaCaT and TIGK cells were plated in a 96-well plate. A 3-(4,5-dimethylthiazol-2-yl)-5-(3-carboxymethoxyphenyl)-2-(4-sulfophenyl)-2H-tetrazolium, inner salt (MTS) proliferation assay was performed to record OD_490_ values corresponding to the numbers of live cells using a spectrophotometer. (**A**) *CLDN1* knockdown in HaCaT and TIGK cell lines. (**B**,**C**) Migration after *CLDN1* knockdown in HaCaT and TIGK cells, respectively. (**D**) Proliferation/viability after *CLDN1* knockdown. (**E**) *OCLN* knockdown in HaCaT and TIGK cell lines. (**F**,**G**) Migration after *OCLN* knockdown in HaCaT and TIGK cells, respectively. (**H**) Proliferation/viability after *OCLN* knockdown. Black = scrambled siRNA, yellow = *CLDN1* siRNA, blue = *OCLN* siRNA; *n* = 3 replicates, * *p* < 0.05.

A recent study by Volksdorf et al. showed that loss of claudin 1 and occludin occurs at the wound margins of chronic wounds and in the regenerating epidermis [16]. On the basis of these results, together with our microarray analyses, we hypothesized that inhibition of *CLDN1* or *OCLN* expression could affect the migratory and proliferative capacity of skin and oral keratinocytes. Small interfering RNA (siRNA) was used to knockdown *CLDN1* or *OCLN* in HaCaT and TIGK cells (Figure 5A,E). In HaCaT cells, *CLDN1* knockdown led to increased migration at 18 h (Figure 5B and Figure S3), whereas *OLCN* knockdown led to decreased migration at 24 h (Figure 5F and Figure S3) compared to scrambled siRNA control. Knockdown of either *CLDN1* (Figure 5D) or *OCLN* (Figure 5H) also led to decreased levels of proliferation/viability in HaCaT cells compared to control. TIGK cells subjected to either *CLDN1* or *OCLN* knockdown showed marginal decreases in migration that were not statistically significant and no significant changes in proliferation/viability were observed compared to the scrambled siRNA control. Although *OCLN* was observed as being highly expressed in both tissues, *OCLN* knockdown only altered the migratory and viability/proliferative capacity of HaCaT cells. These results demonstrate a role for claudin 1 and occludin genes on migration and proliferation/viability in skin keratinocytes, but not oral keratinocytes.

### 2.6. Human Skin and Gingival Keratinocytes Had Distinct Cell Permeability after Histamine Treatment

**Figure 6:**
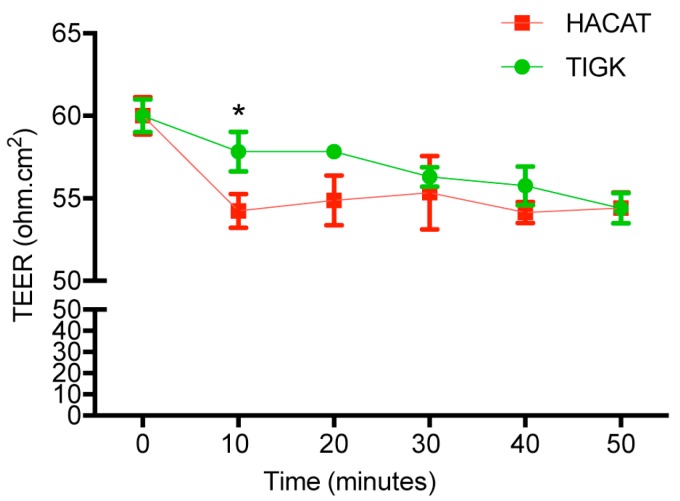
Histamine treatment significantly reduced barrier function in skin keratinocytes. HaCaT and TIGK cells were cultured in 12-well cell culture plates using inserts with pore size of 0.4 µm. At confluency, serum-free DermalLife K Complete medium used in the initial culture with low calcium concentration (0.06 mM) was replaced with high calcium concentration (1.8 mM) DMEM in 10% FBS for 10 days. Cells in the inserts were then treated with histamine (100 µM). The transepithelial electrical resistance (TEER) was measured every 10 minutes. *n* = 3 replicates, * *p* < 0.05.

Histamine is one of the mediators released upon mast cell activation in response to injury [18,19,20]. It improves skin wound healing by enhancing basic fibroblast growth factor production, angiogenesis, and macrophage recruitment [21]. Histamine has been previously shown to disrupt epidermal barriers [22,23]. We hypothesized that histamine treatment would alter the permeability of HaCaT and TIGK cells. We used transepithelial electrical resistance (TEER) to quantitatively measure permeability levels of HaCaT or TIGK cells after histamine treatment (100 µM). After addition of histamine, TEER was reduced in both HaCaT and TIGK cells over the 50 min time course (Figure 6). However, the effect was more rapid in HaCaT cells with a significant decrease in TEER in as early as 10 min. These results suggest that skin keratinocytes are more sensitive to histamine in terms of their barrier function. 

## 3. Discussion

TJs are multiprotein complexes that provide a means of cellular communication, regulation of ion and molecule transport, and maintenance of cell polarity [11,12]. One recent study showed that claudin 1 and occludin are actively present in the regenerating epidermis at the wound edge in acute, but not chronic wounds. This study also showed that inhibition of claudin 1 reduced proliferation and migration of skin keratinocytes [16]. In a human skin suction blister model, water evaporation from the wound was markedly elevated, suggesting impaired epidermal barrier function. However, as these wounds healed, more occludin and ZO1 proteins were present in keratinocytes at the leading edge of keratinocytes, which was accompanied by decreased water evaporation [24]. These results suggest that there is a correlation between wound healing/barrier function and the occludin and ZO1 proteins [24]. In a guinea pig oral hard palate wound model, TJs were absent or fragmentary in the wound epithelium [25]. Multiple studies now suggest that TJs play a critical role in the healing of epithelial tissues (for a comprehensive review of TJ function in wound healing, see [26]). 

The skin and oral mucosa share some histological similarities, yet oral mucosal wounds heal with faster re-epithelialization, decreased inflammation, a refined angiogenic response, and minimal scarring [3,4,5,6,7]. Our most recent studies demonstrate that microRNA 10a/b, 21, and 31 may be partially responsible for the different outcomes observed in skin and oral wound healing [27,28]. Although these findings have been well characterized, the precise mechanisms behind the more regenerative response to injury in the oral cavity is still being elucidated. Because TJ proteins are highly expressed in both skin and oral tissues, and because skin and oral wounds have distinct healing phenotypes, we hypothesized that TJs might play a role in the differential healing phenotypes observed in these tissues. Therefore, the current study investigated the involvement of TJ genes in normal skin and oral tissues and in a wound healing setting.

When comparing the mouse skin epithelium to palate epithelium, we identified *Cldn1* as the most over expressed TJ gene in skin, with *Cldn8* being most over expressed in the palate. High *Cldn1* expression observed in the tail epidermal cell population is consistent with previous reports on this TJ gene as being one of the major transmembrane proteins involved in forming the epidermal paracellular barrier in skin [29]. Interestingly, high *Cldn8* expression has been observed in clinical cancer cell samples relative to benign controls and was shown to promote a pro-migratory and proliferative phenotype in multiple studies [30,31] which is in line with the more proliferative phenotype observed in oral epithelium. Our analysis of the mouse skin and tongue wound healing dataset also identified *Cldn1* and *Ocln*, among other genes, as being differentially expressed in response to injury over the first 5 days in skin but not tongue. The in vitro scratch assay and LPS treatment assay using human gingiva and skin keratinocyte cell lines also identified multiple TJ genes that are differentially expressed in response to injury or LPS stimulation, including *OCLN* in HaCaT cells. Because *CLDN1* and *OCLN* regulate keratinocyte proliferation and migration and because they are lost at the chronic skin wound edges [16], we decided to focus on *CLDN1* and *OCLN* for the remainder of the study. We found that in vitro siRNA knockdown of *CLDN1* caused an increase in migratory capacity and a decrease in proliferation in HaCaT but not TIGK cells. Interestingly, *OCLN* knockdown caused decreased migration and proliferation in HaCaT but not TIGK cells. These results might be expected, as *CLDN1* is significantly more highly expressed at baseline in skin vs. gingiva epithelium due to the role of *CLDN1* in the epidermal paracellular barrier stated above. Taken together, loss of *CLDN1* would likely cause changes in migration and proliferation during the response to injury. 

In our last study, histamine was used to disrupt TJ proteins in HaCaT and TIGK cells and barrier function (TEER) of TJ molecules was measured. These results showed that skin keratinocytes are more sensitive to histamine in terms of their barrier function, exhibiting lower TEER after histamine exposure than oral keratinocytes. We do not know the exact mechanism leading to the decreased TEER by histamine. However, on the basis of other reports, we have a few speculations or theories. First, internalization or endocytosis of TJ molecules [32]—the mechanisms of endocytosis and triggered signaling pathways following endocytosis are rather complicated. The process of endocytosis results in changes to the barrier function and plasticity of TJ molecules. Second, inhibition of epidermal terminal differentiation and disruption of barrier function caused by histamine binding to it receptors—human keratinocytes express H1R, H2R, and H4R [33]. Histamine treatment induces thinning of the epidermis by suppressing epidermal differentiation through inhibition of filaggrin, loricrin, and keratin10 expression in human keratinocytes and in an *ex-vivo* epidermal skin model mediated by H1R [34]. Third, inhibition of expression of TJ molecules such as *ZO1* on epithelial cells by histamine—the effect can be partially abrogated by pretreatment with an H1R antagonist [35]. There is no evidence supporting any of the above theories in this study, thus warranting the need for further studies.

We observed that expression of certain TJ molecules in the unwounded epithelial cell analysis were significantly different from the wound healing tissue expression analysis. For instance, expression of *Ocln* in unwounded mouse skin and 6 h after wounding was significantly higher than the 12–120 h time points, and *Ocln* expression was recovered by 168–240 h (Figure 2). However, the pattern of *Ocln* expression in the scratch assay using HaCaT human keratinocyte cells (Figure 3) seemed to be opposite of what was observed in the wound healing data. There are probably three major reasons behind this. First, one study used mouse skin, whereas the other study used a human keratinocyte cell line. Second, the full thickness dorsal skin wounds include epidermal keratinocytes and the epidermis, dermis, subcutaneous fat, panniculus carnosus, and the interstitial connective tissue. These regions contain many different cell types such as endothelial cells, which have a TJ expression repertoire of their own. Therefore, the expression of a TJ molecule from the skin tissue reflects the level of expression from all types of cells, which also have that particular TJ molecule. Third, due to the multiple cell components of tissues, paracrine signaling affecting TJ expression in skin tissues in vivo is also significantly more complicated than that observed in a single cell line in vitro.

We also note a previous study that showed that inhibition of *CLDN1* reduced proliferation and migration of skin keratinocytes [16], whereas our experiment demonstrated that *CLDN1* knockdown enhanced migration but inhibited proliferation of skin keratinocytes (Figure 5B,D). We believe that there are a few contributing factors to the discrepancy between our current studies and the previous study: (a) keratinocyte cells—we used an immortalized human skin keratinocyte cell line, HaCAT, whereas the other study used primary keratinocyte from human foreskin; (b) migration assay models—we used silicon insert to create gaps as described in the methods without scratching or introducing any trauma to the cells before migration started. The Volksdorf et al. study used pipette tips to scratch confluent cells which causes physical damages to the cells at the edges of the wounds. This physical damage or trauma may cause unexpected functional changes to the cells. Our previous study showed that scratching keratinocytes led to toll-like receptor-4 activation, which resulted in significant inflammatory cytokine tumor necrosis factor-α and interleukin-1β production [36]. We speculate that the inflammatory response from the keratinocytes along with other unknown mechanisms after scratching may have contributed to different cell migration behaviors when *CLDN1* was knocked down.

*CLDN1* is one of the most extensively studied TJ molecules. *CLDN1* knockout mice have wrinkled skin and die within one day after birth. The newborns have significantly increased transepidermal water loss, indicating severely impaired epidermal barrier function [37]. *CLDN1* expression is also markedly decreased in the epidermis of atopic dermatitis patients [22]. Knocking down *CLDN1* significantly inhibited skin keratinocyte proliferation in the current study, which coincides with other studies [16,22]. In addition, *CLDN1* has been shown to be significantly down-regulated at chronic wound edges [16]. These results strongly suggest that *CLDN1* in the epithelium is critical for skin physiology and homeostasis. In the present study, many TJ molecules were found differentially expressed in normal skin and oral tissue or keratinocytes, as well as in wounded tissues during the healing process. However, we do not know how the differential expression of these identified TJ molecules contributes to the different healing phenotypes in skin and oral mucosa. We speculate that these TJ molecules could be differentially regulated in relation to their roles in barrier function, proliferation, or migration of keratinocytes, as well as their roles in innate immunity in the skin and oral mucosa [26]. Further studies are needed to elucidate why there is tissue specificity and the function of these tight junction genes in the wound healing setting.

In summary, we have demonstrated that normal mouse skin and oral epithelia as well as the skin and oral wounds in mouse have significant differential expression of many bicellular TJ genes. Human skin keratinocytes and oral keratinocytes respond differently in their expression of certain TJ genes in response to injury and inflammatory stimulation. Finally, inhibition of claudin 1 or occludin changed the proliferation and migration capacity of human skin but not oral keratinocytes. To the best of our knowledge, this is the first report describing the differential expression of bicellular TJ during skin and oral wound healing. It is clear that the repertoire of TJ genes that the skin and oral tissues use can vary significantly, and regulation of their expression could therefore likely play an important role in wound healing. Our results shed new light on the differential roles of TJ molecules that may contribute to the mechanisms leading to distinct healing phenotypes in skin and oral tissues. However, further investigation of their roles in the wound healing response is needed. Important remaining questions include how the observed differential expression of TJs mechanistically effects the interactions of TJs, the changes of morphology and barrier function of TJs, and the involvement of TJs in keratinocyte proliferation and migration during skin and oral wound healing. 

## 4. Materials and Methods

### 4.1. Animals and Wound Models for Microarray

Although the wound healing and epithelial microarray datasets were generated in prior studies, a brief explanation of the experimental methods used to generate these data is provided for convenience. For the excisional wound healing study, 1 mm full thickness skin and tongue wounds were created on 8-week-old female Balb/c mice (Harlan Inc. Indianapolis, IN, USA). At various intervals after injury (6, 12, 24, 72, 120, 168, and 240 h, *n* = 3 mice per time point per tissue), wounds were harvested and stored in RNAlater (Sigma-Aldrich, St. Louis, MO, USA) [3]. For isolation of skin and oral epithelial cells, full thickness tail skin and the hard palate were collected from 8-week-old female Balb/c mice (Harlan Inc. Indianapolis, IN, USA). Epithelium was separated from the dermis or lamina propria after being treated with 0.2% dispase for 2 h at room temperature, as described in our previous publication (*n* = 4 mice for skin and *n* = 4 mice for palate epithelium) [8]. All animal procedures were approved by the University of Illinois at Chicago Institutional Animal Care and Use Committee (protocol number: ACC 06-091 approved on 19 May 2006 and ACC 09-041 approved on 17 March 2009).

### 4.2. Microarray Data Retrieval and Normalization

The wound healing and epithelial microarray datasets were generated in prior studies [3,8]. Microarray data were downloaded in raw format (.CEL) from the Gene Expression Omnibus (GEO) repository using accession numbers GSE56135 and GSE23006 for the mouse epithelial tissues of skin and hard palate, and the skin and tongue wound healing dataset, respectively. All samples were named according to their names in the GEO repository for clarity. The following processing was performed for each dataset independently. Gene expression arrays were checked for quality control using ArrayAnalysis.org [38], which led to the exclusion of Palate_1 of GSE56135 from further analysis due to large spurious areas in the probe level model (PLM) estimate. Probes were mapped to the custom chip description file (CDF) mouse4302_Mm_ENSG (Version V22.0.0) from brainarray [39], followed by normalization using the robust multichip averaging (RMA) procedure in the R/Bioconductor affy package [40]. 

### 4.3. Microarray Differential Expression Analysis

Differential expression analyses were conducted using the R/Bioconductor software package linear models for microarray data (limma) [41,42]. All comparisons used the moderated *t*-statistic computed for each gene and contrast as described in the limma user guide. For GSE56135 data, a two-group comparison was performed between skin and hard palate epithelia. For GSE23006, skin or tongue response to injury comparisons were conducted by generating contrasts to compare each time point’s gene expression subsequent to wounding to their respective tissue’s baseline expression (time 0 h). For the skin and tongue between group comparison, contrasts were generated to compare skin and tongue at each respective time point without accounting for baseline differences between the two tissues. Significance cutoffs were set at an adjusted *p*-value (*p* < 0.05) using the Benjamini and Hochberg method (BH) to control for false discovery rate (FDR) [43]. All log fold changes (logFC) shown for these analyses are in log_2_ scale. Heatmaps were generated using the heatmap3 package in R [44]. All analyses were performed using R version 3.4.1 [45]. 

### 4.4. Cell Culture

Immortalized human skin keratinocytes (HaCaT) [46] and human telomerase immortalized gingival keratinocytes (TIGK) [47] (ATCC, Manassas, VA, USA) were cultured in 12-well culture plates in serum free DermalLife K Complete Medium (Lifeline Cell Tech LLC, Frederick, MD, USA). For scratch wounding assays, cells were grown to confluency, followed by the creation of three horizontal and three vertical scratch wounds made using the distal edge of a 200 µL pipette tip and removal of any unattached cells via 1× PBS wash and replacement of medium. Cells were harvested with TriZol at 30 min, 4 h, and 24 h after wounding. In another experiment, cells were treated with 100 ng/mL LPS (Sigma-Aldrich) at approximately 80% confluency. Treated cells were harvested with TriZol at 4 h and 24 h after treatment. Cells harvested using TriZol were then stored at −80 °C for total RNA isolation at a later date.

### 4.5. Keratinocyte Migration and Proliferation/Viability after Occludin or Claudin 1 siRNA Knockdown

HaCaT or TIGK cells were grown to 60–70% confluency, then incubated with 10nM of occludin small interfering RNA (siRNA), claudin 1 siRNA, or scrambled control siRNA, complexed with lipofectamine (Life Technologies, Carlsbad, CA, USA) for 72 h. Knockdown efficiency was confirmed by RT-PCR (see results). To examine cell migration, transfected cells were then cultured in 2-well silicone culture inserts with a defined cell-free gap (ibidi, Madison, WI, USA). When cells were confluent outside the gap, culture inserts were removed, and cells were treated with mitomycin C at a concentration of 5 µg/mL (Sigma-Aldrich) for 1 h to inhibit proliferation. After thorough washing to remove mitomycin C, cell migration was then documented by a digital camera at 18 and 24 h. The unfilled gap was then quantified using ImageJ [48]. To examine cell proliferation/viability after transfection, 5 × 10^3^ cells per well were cultured in a 96-well plate. After 24 h, a cell proliferation assay was carried out using a CellTiter 96 Aqueous Non-Radioactive Cell Proliferation Assay kit [a 3-(4,5-dimethylthiazol-2-yl)-5-(3-carboxymethoxyphenyl)-2-(4-sulfophenyl)-2H-tetrazolium, inner salt] (MTS) proliferation assay, Promega, Madison, WI, USA) [49] to determine the relative number of viable cells at OD_490_ by spectrophotometer. 

### 4.6. Real-Time PCR (RT-PCR)

Total RNA from cultured cells was isolated using TriZol (Life Technologies) followed by a DNAse I treatment. For each replicate, 1 µg of total RNA was converted to complementary DNA using the High Capacity Reverse Transcription Kit as per the manufacturer’s instructions (Applied Biosystems, Foster City, CA, USA). mRNA expression of human OCLN, CLDN1, CLDN4, JAM1, JAM3, ZO1, ZO2, and ZO3 were determined by real-time PCR using specific primers (Table S2) and SYBR green PCR mix as per the manufacturer’s instructions (Roche, Basel, Switzerland). Relative expression was calculated using the 2^−∆∆*C*t^ method, with glyceraldehyde 3-phosphate dehydrogenase (GAPDH) used as a house-keeping gene and untreated TIGK used as the baseline. 

### 4.7. TEER Measurement

To examine the barrier function of cultured HaCaT and TIGK cells before and after histamine treatment, cells were cultured in 12-well cell culture plates using Millicell inserts with pore sizes of 0.4 µm (Sigma-Aldrich). When cells reached confluency, serum-free DermalLife K Complete Medium (Lifeline Cell Tech LLC) with low calcium concentration (0.06 mM) was replaced with high calcium concentration (1.8 mM) DMEM in 10% FBS to initiate keratinocyte differentiation and tight junction maturation for 10 days. Inserts containing the confluent differentiated keratinocytes were then transferred to new 12-well plates, and medium with histamine (100 µM) was added, with 1 mL medium in the insert and 2 mL medium into the wells outside of the insert. TEER was measured every 10 min until 50 min after treatment. To measure TEER, we used the chopstick electrode set together with the Epithelial Volt/Ohm (TEER) Meter according to the manufacturer’s instructions (Precision Instruments, Sarasota County, FL, USA).

### 4.8. Statistical Analysis

Results are expressed as means ± standard errors (SE). Wilcoxon test was performed using SAS Version 9.4 (SAS, Cary, NC, USA), and *p*-values less than 0.05 were considered statistically significant. 

## Figures and Tables

**Table 1:** LogFC of differentially expressed genes over time in skin ^1^.

Gene Symbol	6 h	12 h	24 h	72 h	120 h	168 h	240 h
Cldn1	0.41	−0.77	−1.01	−0.61	−0.58	--	--
Cldn2	--	--	0.35	0.37	0.42	0.35	--
Cldn3	--	--	−0.73	−1.07	−0.7	−1.41	--
Cldn4	1	−2.14	−1.79	−1.94	−1.53	−1.03	--
Cldn5	--	--	−0.61	−0.88	--	--	--
Cldn6	--	--	--	--	--	--	--
Cldn7	--	--	--	--	--	--	--
Cldn8	--	−2.8	−2.71	−2.23	−1.44	−1.73	--
Cldn9	--	--	--	--	--	--	--
Cldn10	−0.74	−2.86	−2.74	−2.03	−0.77	−0.62	--
Cldn11	−0.56	−0.49	−0.51	−0.36	−0.38	--	−0.47
Cldn12	--	--	--	--	--	--	--
Cldn13	--	--	--	--	--	0.48	--
Cldn14	--	−0.64	−0.84	−0.91	--	−0.65	--
Cldn15	--	--	--	--	--	--	--
Cldn16	--	--	--	--	--	--	--
Cldn18	--	--	--	--	0.31	--	--
Cldn19	--	--	--	--	--	--	--
Cldn22	--	--	--	--	--	--	--
Cldn23	−1.02	−0.66	−1.54	−1.91	−1.37	−0.82	--
Jam1	0.57	--	--	--	--	--	--
Jam2	−0.47	−0.61	−0.36	--	--	--	--
Jam3	−0.56	--	--	0.45	0.68	0.5	--
Ocln	--	−0.75	−1.41	−0.77	−0.58	−0.61	--
ZO1	0.33	--	−0.46	--	--	--	--
ZO2	0.73	--	−0.53	−0.96	−0.74	−0.65	--
ZO3	−0.75	--	−0.78	--	--	--	--

^1^ Positive values represent gene over expression at that time point vs. baseline. Negative values represent decreased expression at that time point vs. baseline. Cells filled with -- were not statistically significantly differentially expressed.

**Table 2:** LogFC of differentially expressed genes over time in tongue ^1^.

Gene Symbol	6 h	12 h	24 h	72 h	120 h	168 h	240 h
Cldn1	--	--	--	--	--	--	--
Cldn2	--	--	0.39	0.46	0.66	0.76	0.62
Cldn3	--	--	--	--	--	--	--
Cldn4	--	−1.1	−0.97	−1.63	−1.51	--	--
Cldn5	--	--	--	−1.11	−0.84	--	--
Cldn6	--	--	--	--	--	--	--
Cldn7	--	--	--	--	--	--	--
Cldn8	--	--	--	--	--	--	--
Cldn9	--	--	--	--	--	--	--
Cldn10	--	−0.62	--	−0.61	--	--	--
Cldn11	--	--	--	--	--	--	--
Cldn12	--	--	--	--	--	--	--
Cldn13	--	--	--	--	0.47	0.47	--
Cldn14	--	--	--	--	--	--	--
Cldn15	0.72	0.71	0.51	--	--	--	--
Cldn16	--	--	--	--	--	--	--
Cldn18	--	--	--	--	--	--	--
Cldn19	--	--	--	--	--	--	--
Cldn22	--	--	--	--	--	--	--
Cldn23	--	--	--	--	--	--	--
Jam1	--	--	--	−0.41	--	--	−0.56
Jam2	−0.44	−0.64	−0.35	--	--	--	--
Jam3	--	−0.42	--	--	0.43	--	--
Ocln	--	--	--	--	--	−0.52	--
ZO1	--	--	--	--	--	--	--
ZO2	--	--	--	−0.64	--	--	--
ZO3	--	--	--	--	--	--	--

^1^ Positive values represent gene over expression at that time point vs. baseline. Negative values represent decreased expression at that time point vs. baseline. Cells filled with -- were not statistically significantly differentially expressed.

## Supplementary Materials

Supplementary materials can be found at https://www.mdpi.com/1422-0067/21/8/2966/s1.

## Abbreviations

CLDN	claudin
DE	differentially expressed
GAPDH	glyceraldehyde 3-phosphate dehydrogenase
HaCaT	immortalized human skin keratinocytes
JAM	junctional adhesion molecules
LPS	lipopolysaccharide
OCLN	occludin
TEER	transepithelial electrical resistance
TIGK	human telomerase immortalized gingival keratinocytes
TJ	tight junction
ZO	zonula occludens
