# Supplementary material for: Differential Expression and Function of Bicellular Tight Junctions in Skin and Oral Wound Healing
Source: Int J Mol Sci. 2020 Apr 23;21(8):2966. doi: 10.3390/ijms21082966 (PMC7216202; doi:10.3390/ijms21082966)
Supplement: Supplementary file 1 [file ijms-21-02966-s001.pdf]

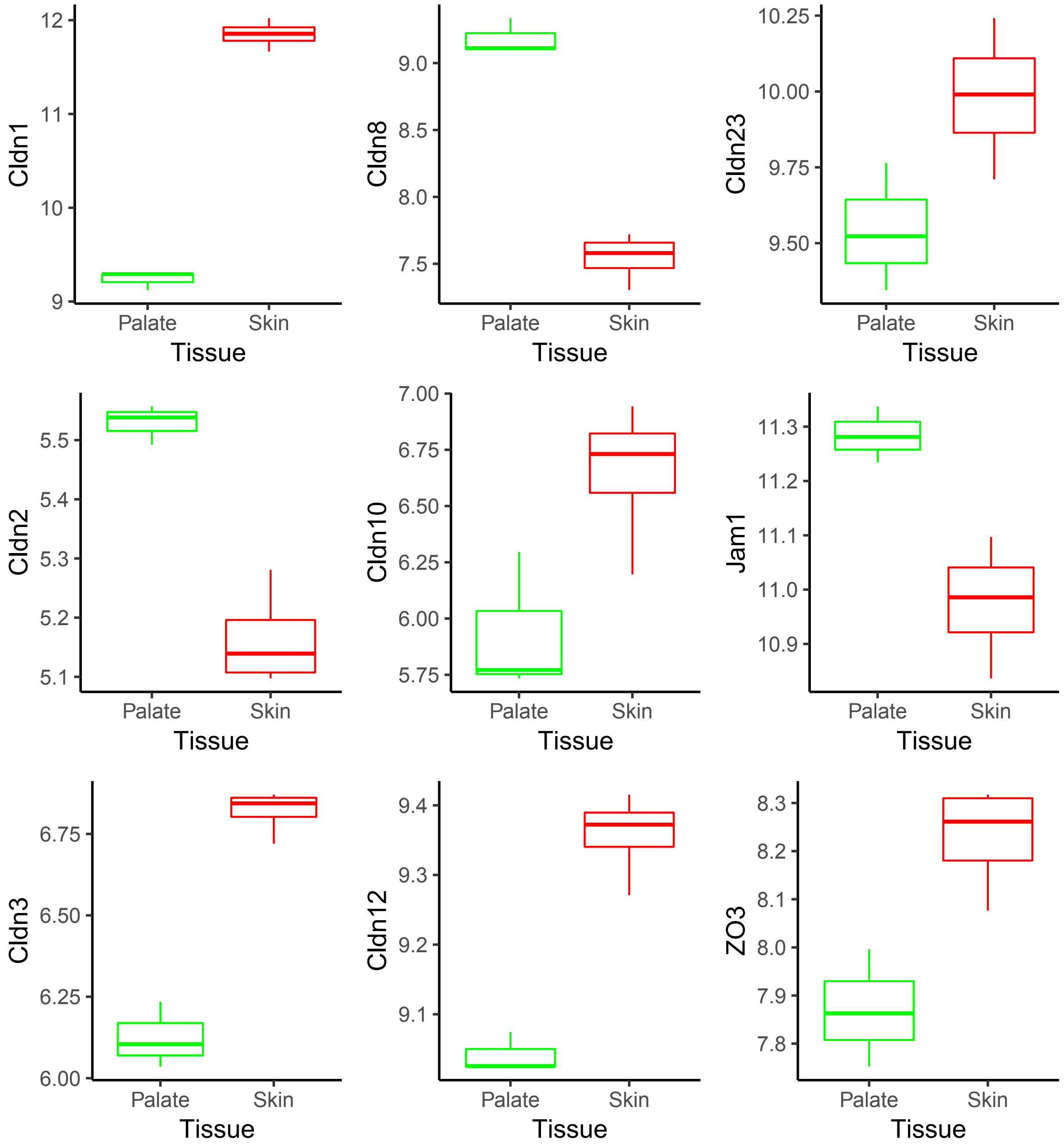

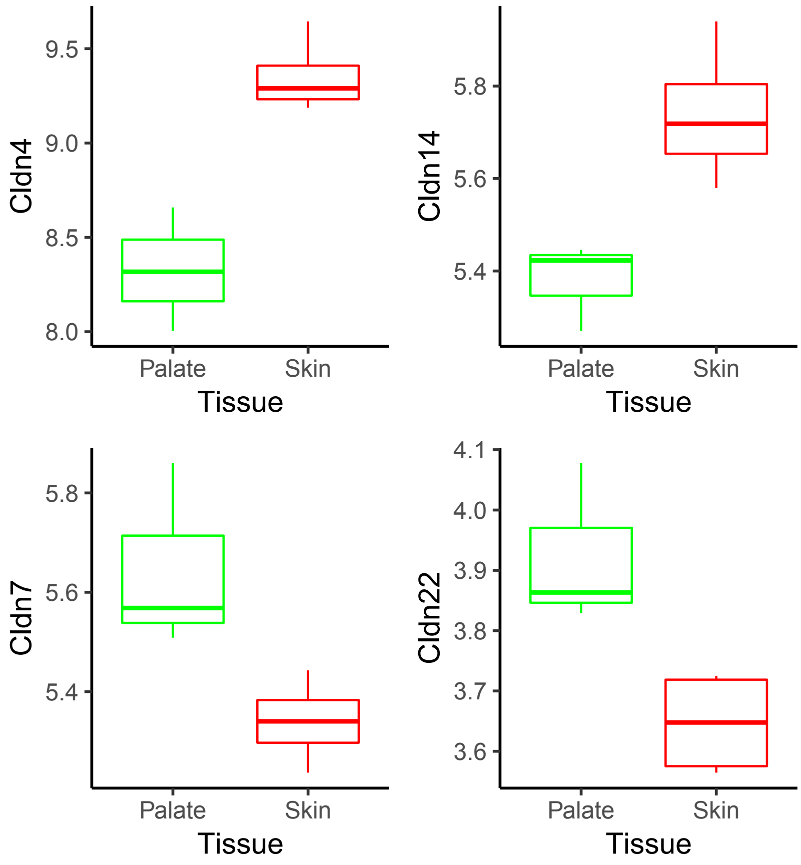

Figure S1. Boxplot of differentially expressed tight junction molecules in primary mouse skin and oral epithelial cells. Skin epithelium obtained from the tails of mice were compared to oral epithelium obtained from the hard palate of mice using LIMMA. Boxplots of each of the 13 differentially expressed TJ genes are presented, using an adjusted p-value of ($q<0.05$) for significance as in Figure 1. Y-axis represents gene expression values in Log2 scale. Green = Palate, Red = Skin.

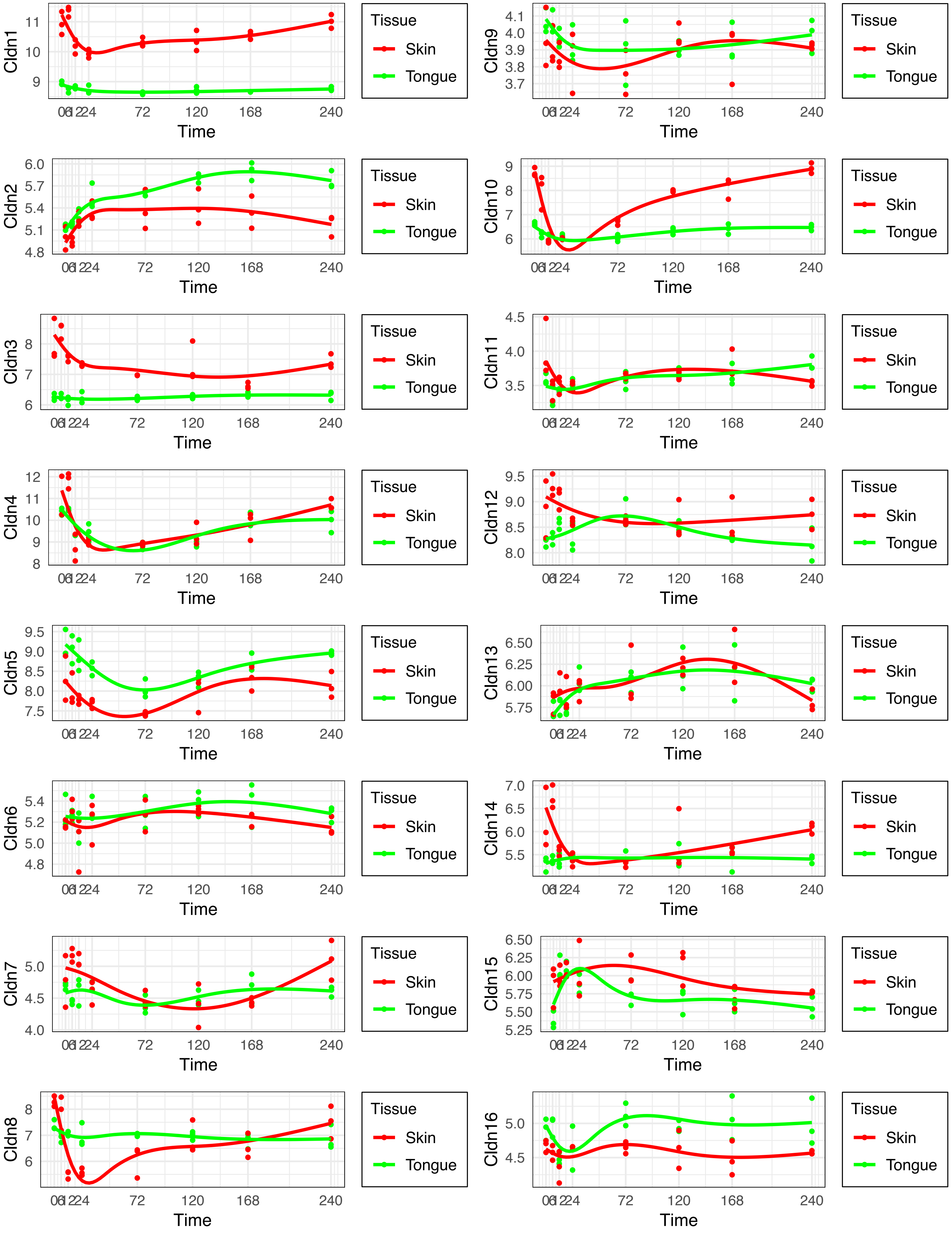

Cldn1
Cldn2
Cldn3
Cldn4
Cldn5
Cldn6
Cldn7
Cldn8
Cldn9
Cldn10
Cldn11
Cldn12
Cldn13
Cldn14
Cldn15
Cldn16
Time
0 6 12 24 72 120 168 240
Tissue
Skin
Tongue

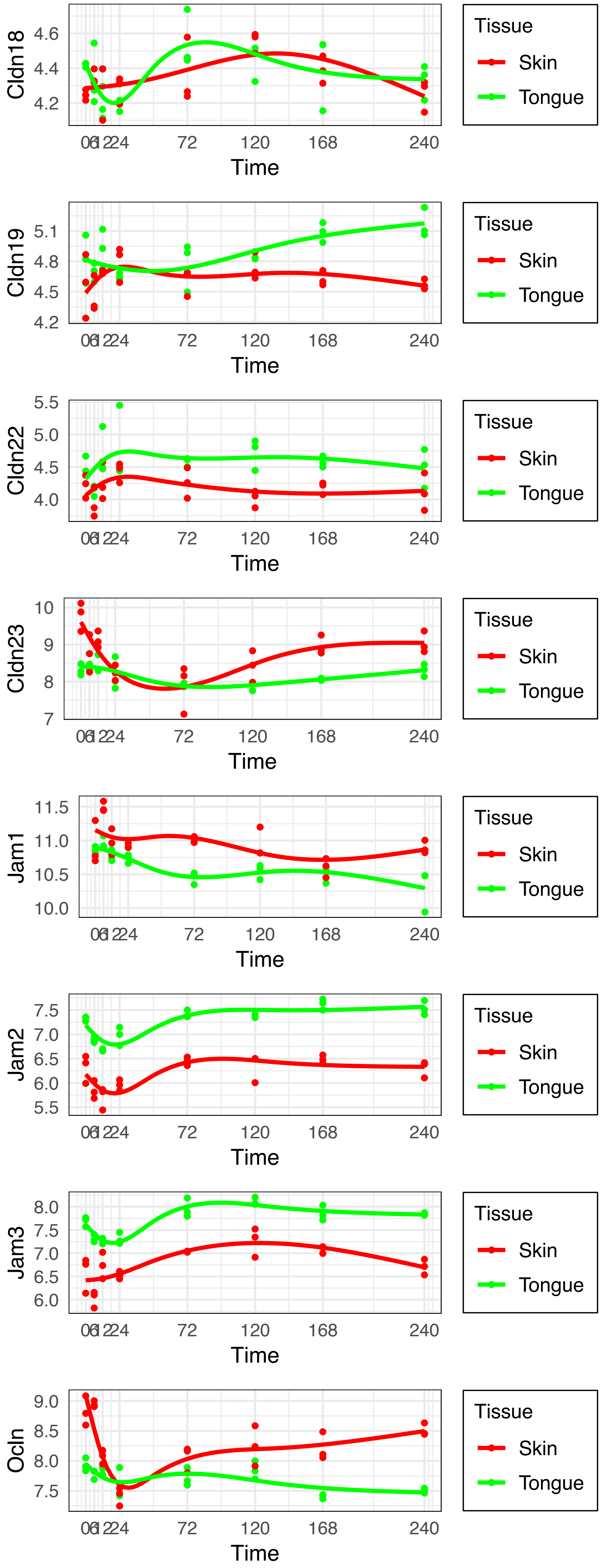

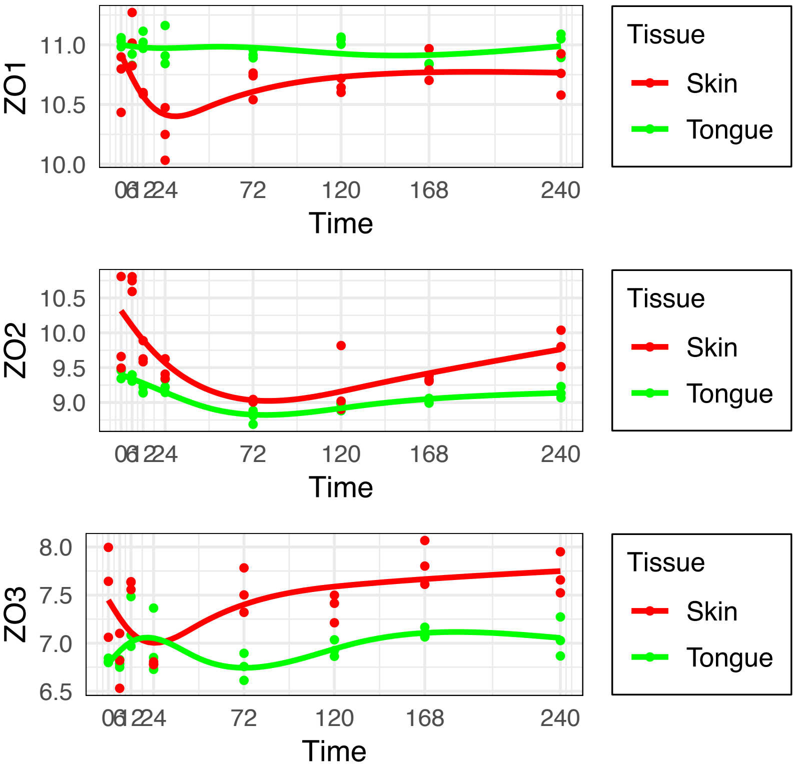

Figure S2. Gene expression of tight junction molecules in mouse skin and oral wounds in vivo. Individual plots displaying the gene expression profiles of the 27 TJ proteins analyzed in response to wounding over 10 days in both skin and tongue wounds. Time 0 represents baseline expression in normal tissue, and subsequent time points represent hours after wounding. A linear model with formula = y~ns (x,4) was used to create the line plots, standard error is not displayed. Each dot represents a sample, Green = Tongue, Red = Skin, X-axis = Time (hours), Y-axis = gene expression values in log2 scale.

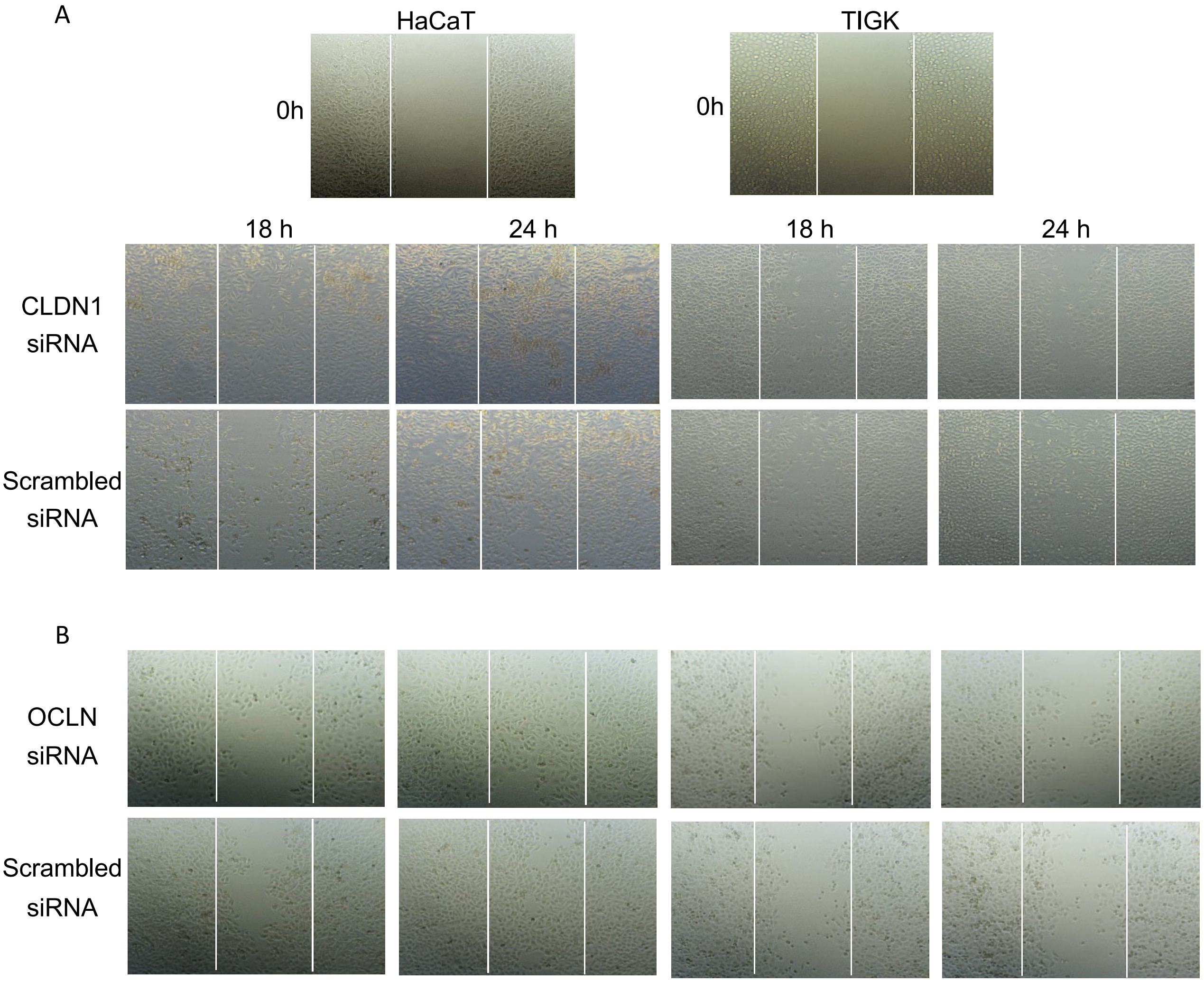

Figure S3. Representative images of migration of skin and oral keratinocytes after CLDN1 or OCLN knock down from data presented in Figure 5. HaCaT cells are in the left panel and TIGK are in the right panel. Migration after A) CLDN1 or B) OCLN knock down.

Table S1. LogFC of differentially expressed genes over time between tongue vs skin.[1]

Gene Symbol	0 h	6 h	12 h	24 h	72 h	120 h	168 h
Cldn1	-1.99	-2.62	-1.35	-1.21	-1.71	-1.63	-1.9
Cldn2	--	--	--	--	--	0.4	0.57
Cldn3	-1.78	-2.18	-1.41	-1.08	-0.75	-1.09	--
Cldn4	--	-1.36	--	--	--	--	--
Cldn5	0.85	1.06	1.08	0.87	0.63	--	--
Cldn6	--	--	--	--	--	--	--
Cldn7	--	-0.73	-0.46	--	--	--	--
Cldn8	-0.92	-0.96	1.59	1.37	0.96	--	--
Cldn9	--	--	--	--	--	--	--
Cldn10	-2.12	-1.81	--	--	-0.69	-1.62	-1.71
Cldn11	-0.42	--	--	--	--	--	--
Cldn12	-0.66	-1.03	-0.51	--	--	--	--
Cldn13	--	--	--	--	--	--	--
Cldn14	-0.92	-1.35	--	--	--	--	--
Cldn15	-0.51	--	--	--	--	-0.48	--
Cldn16	--	0.4	--	--	0.48	--	0.59
Cldn18	--	--	--	--	--	--	--
Cldn19	--	--	--	--	--	--	0.46
Cldn22	--	--	--	--	--	0.7	--
Cldn23	-1.47	--	-0.68	--	--	--	-0.91
Jam1	--	-0.53	--	--	-0.55	-0.4	--
Jam2	1.01	1.04	0.97	1.02	0.96	1.03	1.14
Jam3	1.1	1.29	0.53	0.78	0.93	0.85	0.77
Ocln	-0.89	-1.16	--	--	--	-0.4	-0.81
ZO1	0.31	--	0.45	0.72	--	0.38	--
ZO2	-0.56	-1.37	-0.53	--	--	--	--
ZO3	-0.75	--	-0.43	--	-0.78	-0.43	-0.72

[1]Positive values represent gene over expression in tongue at that time point vs. baseline. Negative values represent over expression in skin at that time point vs. baseline. Cells filled with -- were not statistically significantly differentially expressed (DE).

Table S2. Primer sequences for Real-Time PCR

Target Genes	Forward Primer (5′ ---- 3′)	Reverse Primer (5′ ---- 3′)
CLDN1	CCAGTTAGAAGAGGTAGTGTGAAT	CAGCCAGCTGAGCAAATAAAG
CLDN4	GCCTTACTCCGCCAAGTATT	AGGGAAGAACAAAGCAGAGAG
JAM1	ACCTGGTTCAAAGATGGGATAG	TGTTGTGGGATTCAGGACATAG
JAM2	AAGTTAGTGCCCCATCTGAG	GGATTCCCTTCTTTGTCTTG
JAM3	GGGCTGTAAATCTCAAATCC	TCTCTCCGTGTCACATTCC
OCLN	CCCATCTGACTATGTGGAAAGAG	AACCGGCGTGGATTTATAGG
ZO1	CCTGAGTTTGACAGTGGAGTT	GCTGAAGGACTCACAGGAATAG
ZO2	GGAAGGTCGCTGCTATTGTG	CGGAAACTTCTGCCATCAAAC
ZO3	GAGACAGCGAAGAGTTTGG	TAGACACCCCGTTGATCTG
GAPDH	CAGGGCTGCTTTTAACTCTGG	TGGGTGGAATCATATTGGAACA
